# Pregnancy-associated plasma protein A mRNA expression as a marker for differentiated thyroid cancer: results from a “surgical” and a “cytological” series

**DOI:** 10.1007/s40618-021-01655-9

**Published:** 2021-08-04

**Authors:** C. Marzocchi, M. Capezzone, A. Sagnella, A. Cartocci, M. Caroli Costantini, L. Brindisi, V. Mancini, S. Cantara, M. G. Castagna

**Affiliations:** 1 Department of Medical, Surgical and Neurological Sciences, University of Siena, Viale Bracci 16, 53100 Siena, Italy; 2 Department of Medical Biotechnologies, University of Siena, Siena, Italy; 3 Department of Oncology and Pathological Anatomy, Azienda Ospedaliera, Universitario Senese, Siena, Italy

**Keywords:** Pregnancy-associated plasma protein A, Thyroid cancer, IGF pathway, FNAC, Tumor marker

## Abstract

**Purpose:**

Pregnancy-associated plasma protein A (PAPPA) is a metalloproteinase initially described for its role during pregnancy. PAPPA regulates IGF ligands 1 (IGF1) bioavailability through the degradation of IGF-binding protein 4 (IGFBP4). After the cleavage of IGFBP4, free IGF1 is able to bind IGF1 receptors (IGF1R) triggering the downstream signaling. Recently, PAPPA expression has been linked with development of several cancers. No data have been published on thyroid cancer, yet.

**Methods:**

We evaluated PAPPA, insulin-like growth factor (IGF1), IGF1 receptors (IGF1R) and IGF-binding protein 4 (IGFBP4) mRNA expression levels in a “Surgical series” of 94 thyroid nodules (64 cancers, 16 follicular adenomas and 14 hyperplastic nodules) and in a “Cytological series” of 80 nodules from 74 patients underwent to fine-needle aspiration cytology (FNAC). In tissues, PAPPA was also evaluated by western blot.

**Results:**

We found that PAPPA expression was increased in thyroid cancer specimen at mRNA and protein levels and that, adenomas and hyperplastic nodules had an expression similar to normal tissues. When applied on thyroid cytologies, PAPPA expression was able to discriminate benign from malignant nodules contributing to pre-surgical classification of the nodules. We calculated a cut-off with a good specificity (91%) which reached 100% when combined with molecular biology.

**Conclusion:**

These results show that PAPPA could represent a promising diagnostic marker for differentiated thyroid cancer.

## Introduction

Pregnancy-associated plasma protein A (PAPPA), a metalloproteinase of 1627 amino acids [1], represents a key regulation factor of insulin-like growth factor (IGF) pathway. PAPPA, binding proteoglycans on the cell surface, regulates IGF ligands 1 (IGF1) bioavailability through the degradation of IGF-binding protein 4 (IGFBP4), which represents its principal substrate. After the cleavage of IGFBP4, free IGF1 is able to bind IGF1 receptors (IGF1R) triggering the downstream signaling [2]. Circulating high levels of PAPPA were discovered in pregnant women but were also expressed widely in different tissues and fluids [3]. As well as in pregnancy, an increased expression has been found in pathophysiological conditions as cardiovascular disease [4] and inflammatory states [5] and its role has been studied in different diseases, such as diabetes, obesity and cancer [3]. In literature, the involvement of PAPPA in cancer development and progression has been explored in multiple cancer types supporting its possible role as oncogene. PAPPA is overexpressed in ovarian cancer [6], lung cancer [7], breast cancer [8], Ewing sarcoma [9], testicular and prostate cancer [10] and hepatocellular carcinoma [11]. Moreover, evidences suggest that PAPPA activity promotes cancer proliferation, invasion, migration and metastasis [12–15]. In literature, data are missing about the relationship between PAPPA and thyroid cancer, although the IGF axis has been actively investigated in thyroid tumorigenesis [16]. Only one study [17] reports increased PAPPA mRNA levels in thyroid malignancies using online gene profiling data. However, authors have analyzed PAPPA levels in a small number of thyroid cancers (*n* = 26) and compared to only 4 non-tumor tissues [17].

In our study, we aimed to characterize the expression levels of PAPPA and other components of the IGF system in a wider number of thyroid cancer specimen and compare the results with mRNA levels in adjacent healthy tissues as well as in hyperplastic nodules and adenomas. Furthermore, we investigated the potential diagnostic role of PAPPA to discriminate benign and malignant thyroid nodules in cytological material obtained after fine-needle aspiration.

## Materials and methods

### Patients

A retrospective analysis was conducted in two independent cohort of samples: “surgical series” and “cytological series”. The “surgical series” was composed by 94 consecutive tissues of thyroid nodules and their healthy adjacent thyroid tissues belonging to 94 patients with thyroid nodular disease followed in the section of Endocrinology (University of Siena, Italy) and submitted to total thyroidectomy from 2015 to 2020. The study cohort included 29 males (30.8%) and 65 females (69.2%), with an average age at diagnosis of 47.2 ± 14.4 years (range 17–82 years). At final histology, a total of 64/94 (68%) cancers were found. They were 61 (95.3%) papillary thyroid cancers (PTC), 1 (1.6%) follicular thyroid cancer (FTC) and 2 (3.1%) undifferentiated thyroid cancers. In the remaining 30 cases, 16/30 (53.4%) were follicular adenomas and 14/30 (46.6%) were hyperplastic nodules. The clinical–pathological features of thyroid cancer patients are summarized in Table 1. The “cytological series” was an independent consecutive series represented by a total of 80 thyroid nodules belonging to 74 patients underwent to fine-needle aspiration cytology (FNAC) under ultrasound guidance from 2015 to 2020 and of which the cytological material was still available in our tissue bank. This cohort of patients included 19 males (25.6%) and 55 females (74.4%), with an average age at diagnosis of 49.7 ± 14.6 years (range 13–77 years). FNAC's results were classified according to Bethesda classification [18]. 42/80 (52.5%) were Bethesda II, 31/80 (38.7%) were Bethesda categories III–IV and 7/80 (8.7%) were Bethesda V–VI. All patients belonging to Bethesda III–IV and V–VI groups underwent surgery. At final histology, 15/31 (48.4%) Bethesda III–IV were follicular adenomas and 16/31 (51.6%) were found to be malignant (3 CPT, 6 CF, 7 CPTVF). All Bethesda V–VI studied were confirmed to be malignant (PTC). Patients signed an informed consent and the study was approved from local Ethical Committee (Comitato Etico Regione Toscana, Area Vasta Sud Est, AOUS. Protocol ID: 10167).

**Table 1:** Clinical–pathological characteristics of 64 patients with thyroid cancer belonging to the “surgical series”

Parameters	*n* (%)
Sex	
Males	21 (32.8)
Females	43 (67.2)
Multi-focality	
Yes	27 (42.2)
Not	37 (57.8)
Bi-laterality	
Yes	17 (26.6)
Not	47 (73.4)
Lymph node metastases	
Yes	25 (39)
Not	39 (51)
Extra-thyroidal invasion	
Yes	29 (45.3)
Not	35 (54.7)
Variants of PTC°	
Classic	36 (59)
Follicular	9 (14.7)
Tall cell	4 (6.5)
Columnar	3 (4.9)
Sclerosing	8 (13.1)
Warthin-like	1 (1.8)
Tumor size diameter (cm)	1.93 ± 1.24
Age at diagnosis	46.5 ± 15.7

°Available on 61 cases

*Mean ± standard deviation

### RNA isolation from tissues and cytological material

Pathological thyroid tissues and adjacent healthy tissues collected at surgery were immediately stored at − 80 °C. Frozen tissues were disrupted and homogenized in RLT lysis buffer (Qiagen) with the T8-Ultra-Turrax (Ika-Werke, Staufen, Germany) homogenizer. Total RNA was extracted from supernatant using the RNeasy minikit (Qiagen) following kit instructions. Cytological material obtained after fine-needle aspiration cytology procedure from thyroid nodules was conserved in RNAprotect Cell Reagent (Qiagen) and RNA isolation was carried out always using RNeasy minikit (Qiagen). After quantification and purity assessment (Abs260/280) with NanoDrop One RNA (Thermo Scientific), 200 ng for each sample was retro-transcribed with M-MuLV-RH First Stand cDNA Synthesis Kit (Experteam).

### Quantitative real-time PCR

Quantitative real-time polymerase chain reaction (qRT-PCR) was carried out with Rotor-Gene Q real-time PCR system (Qiagen) using FastStart Essential DNA Green Master Roche for PAPPA and IGF1R gene expression. Specific primers were: PAPPA PF: 5′-TGAATCTGAGCAGCACATTG-3′ and PR: 5′-CATCGTCTTCCAAGCACTTC-3′; IGF1R PF: 5′-GCCACATCTCTCTCTGGGAA-3′ and PR: 5′-GGAACGTACACATCAGCAGC-3′. Applied Biosystems' Taqman gene expression assays were used for IGF1 (Hs01547656_m1) and IGFBP4 (Hs01057900_m1). Samples were normalized to GAPDH or ACTB and quantification was determined using the 2^−ΔCT^ method. Each sample was run in triplicate.

### Western Blot

Proteins were extracted from 12 tissues (6 PTCs and 6 healthy controlateral parts). Tissues were weighted and lysed using the CelLytic™ M reagent (Sigma-Aldrich). For each gram of tissue, 20 ml of reagent was added. After homogenization, tissues were centrifuged at 13,000×*g* for 10 min. Proteins contained in the supernatant were quantified by Bradford using a standard curve prepared with BSA (stock concentration 2 mg/ml) composed of five dilution points (250, 125, 50, 25 and 5 µg/ml).

50 mg of proteins will be mixed with 4X reducing SDS-PAGE sample buffer and denatured at 100 °C for 10 min. Electrophoresis was carried out in SDS/10% polyacrylamide gel at 150 V. Proteins were then blotted onto activated PVDF membranes at 390 mA for 90 min. Aspecific sites were saturated overnight with 10 ml of PBS/Tween containing 10% non-fat dry milk (Biorad). After, membranes were incubated overnight with primary PAPP-A antibody diluted 1:200 (Santa Cruz) at 4 °C, washed in PBS/Tween and incubated 1 h at room temperature with secondary antibody. Signals were detected by enhanced chemiluminescence system (Amersham). Results were normalized against GAPDH (Cell Signaling; dilution 1:1000). OD arbitrary units were calculated using the ImageJ software subtracting the background from each measures.

### Mutation analysis

For BRAF point mutation (V600E), cDNA was amplified using 2× AmpliTaq Gold PCR master mix (Applied Biosystems Inc., Milano, Italy) with a final primer concentration of 200 nM at 60 °C (35 cycles). RAS mutations (H-, K-, and N-RAS, codons 12 and 61), were analyzed by PCR with 200 nM primer final concentration at 64.9 °C for H-RAS and 61 °C or K-RAS and N-RAS. RET/PTC1 and RET/PTC3 re-arrangements were analyzed by qRT-PCR [19]. PAX8/PPArgamma rearrangements were evaluated as previously described [20]. Specific primers are available upon request.

### Statistical analysis

A minimum sample size of 62 was estimated, based on two-sided Mann–Whitney test, considering first type error of 0.5, power of 0.80 and a large effect size of 0.75.

Statistical analysis was performed using GraphPad Prism software version 5 or Statview v.5.0.1 for clinical variable evaluation. Differences in mRNA expression between pathological thyroid tissues (cancers, hyperplastic nodules and adenomas) and their adjacent healthy thyroid tissues were evaluated by Wilcoxon signed-rank test. The Mann–Whitney *U* test was carried out to compare outcomes between two independent groups. The Kruskal–Wallis test, followed by Dunn's test in case of significance, was used to compare three or more independent groups. For the association of PAPPA levels and age at diagnosis and tumor size, Spearman’s correlation was used. Diagnostic accuracy was evaluated by the area under the ROC curve (AUC) and its 95% confidence interval (CI). The best cut-off value was calculated with Youden index providing the best tradeoff between sensitivity and specificity. For all comparisons, a *p* value < 0.05 was considered significant.

## Results

### PAPPA expression in thyroid specimen

**Fig. 1:**
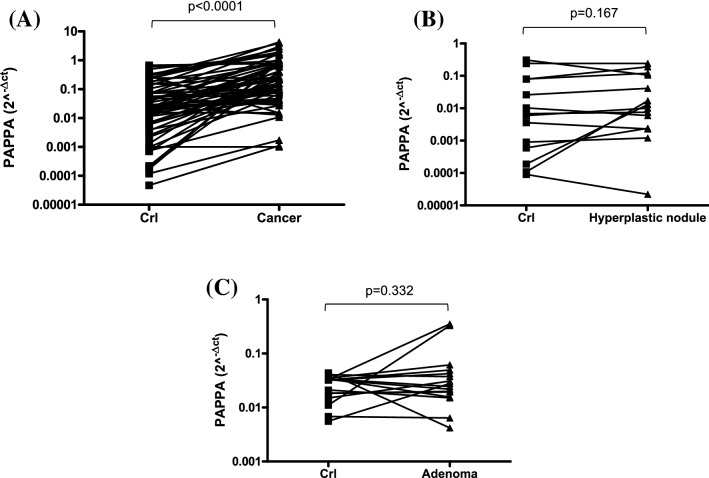
Expression of PAPPA in cancer tissue (A; *n* = 64), hyperplastic nodules (B; *n* = 14) and adenoma (C; *n* = 16) and corresponding healthy tissues by real-time PCR. Results are reported as 2^−ΔCT^. Expression levels were significantly different only between cancers and the controlateral healthy counterparts with a *p* < 0.0001 by Wilcoxon signed-rank test

**Fig. 2:**
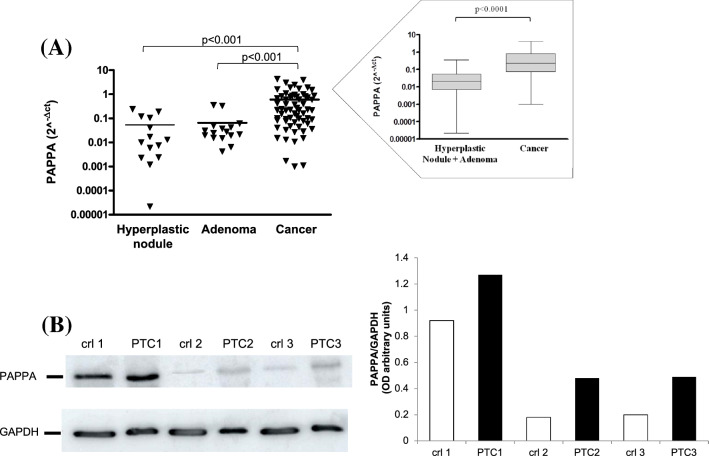
**A** PAPPA mRNA expression calculated with real-time PCR and reported as 2^−ΔCT^ in thyroid cancers (*n* = 64) compared to hyperplastic nodules (*n* = 14) and adenomas (*n* = 16). *p* < 0.001 by Kruskal–Wallis test with Dunn's Multiple comparison test. In the box, results for PAPPA expression for benign pathologies (*n* = 30) have been grouped and compared with cancers (*n* = 64), *p* < 0.0001 by Mann–Whitney *U* test. **B** Representative gel of three PTCs and their adjacent healthy tissue and its quantification by ImageJ

PAPPA mRNA expression measured by qRT-PCR was significantly higher (*p* < 0.0001) in 64 thyroid cancers compared to their benign counterparts (Fig. 1A). No difference was found in hyperplastic nodules (*n* = 14) and adenomas (*n* = 16) when compared to the corresponding normal thyroid (*p* = 0.167 and *p* = 0.332, respectively) (Fig. 1B, C). PAPPA mRNA expression was higher in thyroid cancer compared to hyperplastic nodules and adenomas (*p* < 0.001) (Fig. 2A) and this difference was more evident when we group the benign pathologies and compared the results with cancer (*p* < 0.0001) (Fig. 2A, box).

When evaluated for mutation, 77.7% of cancers had BRAF V600E; 8.8% showed RET/PTC rearrangements; 4.4% were positive for PAX8/PPAr gamma and 9.1% had RAS mutations (H- and K-, no NRAS mutations were found in our series). Due to the high prevalence of BRAF mutation, no association was found between PAPPA expression and mutational status (*p* > 0.5).

PAPP-A was also evaluated by western blot in a small cohort of tissues and in particular in 6 PTCs and their controlateral parts. Figure 2B shows a representative gel and its quantification. As observed, PTCs had an increased expression of PAPP-A also at protein level compared to adjacent healthy tissue.

### Correlation between PAPPA expression clinical–pathological features in thyroid cancer patient (*n* = 64)

In the surgical series, within the thyroid cancer group (*n* = 64), we did not observe any statistical significant difference according to histological variant (*p* = 0.5), extra-thyroidal invasion (*p* = 0.3), bi-laterality (*p* = 0.1), multi-focality (*p* = 0.9), rate of lymph node metastases (*p* = 0.1) and sex (*p* = 0.2) (Table 2). In addition, no correlation was found between PAPPA expression and age at diagnosis (*p* = 0.5) or tumor diameter (*p* = 0.6) (Table 2).

**Table 2:** Correlation between PAPPA expression and clinical–pathological features in 64 patients with thyroid cancer belonging to the “surgical series”

Parameters	PAPPA	*p*
Sex		
Males	0.592 ± 0.9	0.2*
Females	0.627 ± 1.0	
Multi-focality		
Yes	0.690 ± 1.1	0.9*
Not	0.561 ± 0.6	
Bi-laterality		
Yes	1.075 ± 1.4	0.1*
Not	0.467 ± 0.6	
Lymph node metastases		
Yes	0.741 ± 0.9	0.1*
Not	0.535 ± 0.8	
Extra-thyroidal invasion		
Yes	0.596 ± 0.8	0.3*
Not	0.631 ± 0.9	
Variants of PTC°		
Classic	0.656 ± 0.9	0.5**
Follicular	0.856 ± 0.9	
Tall cell	0.191 ± 0.06	
Columnar	0.106 ± 0.04	
Sclerosing	0.441 ± 0.6	
Warthin-like	1.500 ± 0.0	
Tumor size	–	0.6***
Age at diagnosis	–	0.5***

*PTC* papillary thyroid cancer

*By Mann–Whitney test

**By Kruskal–Wallis

***By Spearman’s correlation

°Available on 61 cases

### PAPPA expression in cytological sample

**Fig. 3:**
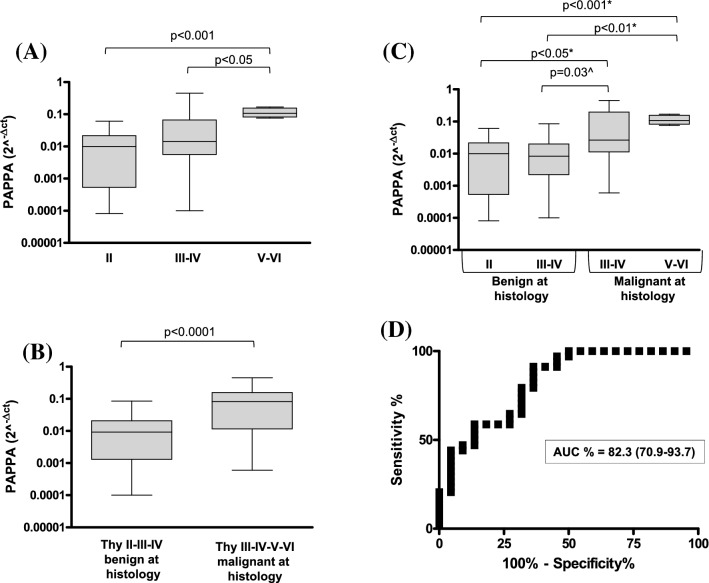
PAPPA mRNA expression in 80 FNAC **A** 42 Bethesda II, 31 Bethesda III–IV and 7 Bethesda V–VI. *p* < 0.05 and *p* < 0.001 by Kruskal–Wallis test with Dunn's Multiple comparison test. **B** Results for PAPPA expression for nodules benign at histology (*n* = 57) and compared with nodules malignant at histology (*n* = 23), whatever the cytological category. *p* < 0.0001 by Mann–Whitney *U* test. **C** PAPPA mRNA expression stratified according with the FNAC category and final histology of the nodules. *p < 0.05, *p* < 0.01 and *p* < 0.001 by Kruskal–Wallis test with Dunn's Multiple comparison test. ^*p* = 0.03 considering only the indeterminate cytology and applying a Mann–Whitney *U* test between Bethesda III–IV benign at histology and Bethesda III–IV malignant at histology. **D** Diagnostic accuracy of PAPPA mRNA expression in FNAC evaluated by the area under the ROC curve (AUC) and its 95% confidence interval (CI)

We analyzed a total of 80 FNAC. As shown in the Fig. 3A, Bethesda V–VI (*n* = 7) category displayed a statistically significant higher expression of PAPPA compared to II (*n* = 42) and III–IV (*n* = 31) categories (*p* < 0.01 and *p* < 0.05, respectively). Although no difference was found between categories III–IV compared to Bethesda II, the increasing trend observed in Fig. 3A was statistically significant applying a Youden cut-off of 0.0302. This effect was more evident by analyzing cytological categories only according with the final histology (Fig. 3B, *p* < 0.0001).

PAPPA expression was also evaluated in the subgroup of Bethesda III–IV nodules according to final histology. In malignant Bethesda III–IV nodules (*n* = 16), PAPPA expression was similar to Bethesda V–VI (*p* = 0.20) while it was increased when compared to Bethesda II nodules (*p* < 0.05). Similarly, benign Bethesda III–IV nodules at histology (*n* = 15) showed similar PAPPA expression compared with Bethesda II (*p* = 0.42) and lower PAPPA expression compared with Bethesda V–VI nodules (*p* < 0.01) (Fig. 3C). Again, stratifying expression results only for indeterminate cytology and applying a Mann–Whitney *U* test, we observed a statistically significant difference (*p* = 0.03) between Bethesda III–IV malignant and benign at histology (Fig. 3C).

Generally, according also with the European Thyroid Association guidelines [19], cytologically indeterminate samples can undergo molecular analysis to drive clinical decision. We applied the seven gene panel [19] to Bethesda categories III–IV (*n* = 31) samples and we found 2 false positive (FP) and 4 false negative (FN) results according to final histology [sensitivity 71.4%, specificity 88.2%, positive predictive value (PPV) 83.4% and negative predictive value (NPV) 78.9%]. Hypothesizing to use PAPPA expression in FNAC to discriminate benign from malignant nodules, we calculated the ROC curve together with its 95% CI considering all cytological samples (Fig. 3D). The area under ROC curve, AUC, demonstrates a moderate degree of diagnostic accuracy (AUC% = 82.3%, 95% CI 70.9–93.7%). The calculated Youden cut-off of 0.0302 showed a specificity of 91%, sensitivity of 63% with an accuracy of 81%, PPV of 80% and NPV of 82%. Combining together molecular biology and PAPPA expression, we correctly classified all benign lesions and obtained only 2 FN results with a final sensitivity of 85.7%, specificity 100%, PPV 100% and NPV 89.5%.

### Analysis of the IGF1 cascade in thyroid specimen

**Fig. 4:**
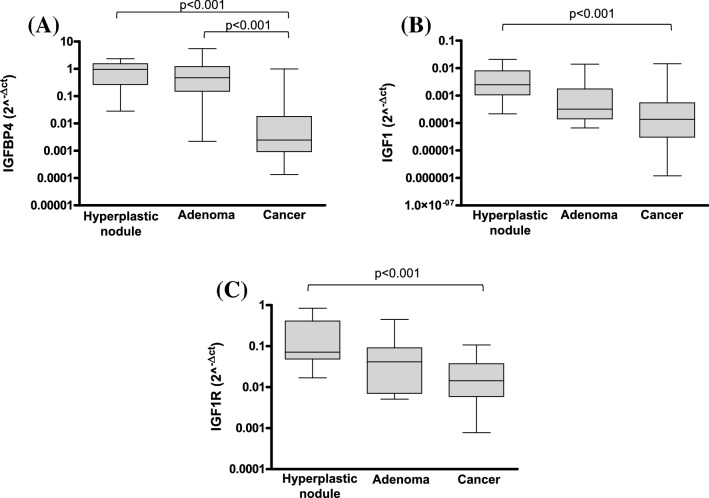
IGFBP4 (**A**), IGF1 (**B**) and IGF1R (**C**) mRNA expression levels calculated by real-time PCR and reported as 2^−ΔCT^ in hyperplastic nodules (*n* = 14), adenomas (*n* = 16) and cancer (*n* = 64) tissues. *p* < 0.001 by Kruskal–Wallis *H* test with Dunn's Multiple comparison test

PAPPA activities are linked to IGFBP4 cleavage that leads to the separation of IGF1 from the IGFBP4-IGF1 complex with an increase in free IGF1 that can bind to IGF1R. We evaluated mRNA expression of IGFBP4, IGF1 and IGF1R in thyroid cancers, adenomas and hyperplastic nodules in comparison with their benign counterparts without observing any statistically significant differences (Table 3). On the contrary, as illustrated in Fig. 4, we observed a significantly lower level of IGFBP4 in cancers compared with both hyperplastic nodules and adenomas (*p* < 0.001), while no differences were found between the two groups of benign lesions (Fig. 4A). Similar results were observed for IGF1 (Fig. 4B) and IGF1R (Fig. 4C).

**Table 3:** Correlation of IGFBP4, IGF1 and IGF1R levels between the group of hyperplastic nodules, adenomas and cancers and the corresponding adjacent non-tumor tissue group

Group	mRNA expression	p*
Hyperplastic nodules vs healthy adjacent tissues	IGFBP4	0.06
	IGF1	0.06
	IGF1R	0.8
Adenomas vs healthy adjacent tissues	IGFBP4	0.1
	IGF1	0.06
	IGF1R	0.6
Cancers vs healthy adjacent tissues	IGFBP4	0.6
	IGF1	0.2
	IGF1R	0.1

*By Mann–Whitney test

### Discussion

High expression levels of PAPPA, a metalloproteinase that regulates IGF bioavailability in vitro through cleavage of inhibitory IGFBP-4, have been found in several malignancies [17]. Its pro-tumor functions include promotion of cancer cell proliferation, invasion, migration and metastasis [17]. These actions can be linked to IGF1 release [15, 21, 22] or can be independent from IGFBP-4 proteolysis [6].

This is the first report showing elevated PAPPA mRNA expression in a robust series of thyroid cancer specimen (*n* = 64) comparing with normal thyroid counterparts and benign thyroid lesions (14 hyperplastic nodules and 16 adenomas). PAPPA expression levels were not associated with mutational status, histological variants and clinical features of thyroid cancer suggesting that PAPPA expression could not have a role as prognostic factor in thyroid cancer. However, to drive definite conclusions on the correlation between PAPPA expression and clinical features and consequently its possible role as prognostic marker, more samples need to be analyzed.

Since the results on thyroid tumor were robust and reproducible also at protein level, we investigated the possibility to analyze PAPPA expression in cytological samples as diagnostic marker to discriminate benign from malignant nodules. It is widely accepted that fine-needle aspiration cytology (FNAC) represents the gold standard to define the nature of thyroid nodules. FNAC is a reliable method with good sensitivity and specificity [23]. The “dark side of the moon” of FNAC is represented by indeterminate lesions [atypia of undetermined significance/follicular lesion of undetermined significance (Bethesda III) and follicular neoplasm/suspicious for follicular neoplasm (Bethesda IV)] and researchers have contributed molecular markers to be search for in cytological material to improve FNAC diagnosis [24–27]. Together with classical mutations (including at least BRAF, RAS, hTERT point mutations and RET/PTC, NTRK, PAX8/PPARγ rearrangements), other tools have been included in clinical practice, such as Next-Generation Sequencing panels [25, 26] and Gene Expression Classifier [27], and others markers have been only hypothesized such as the use of miRNA [28, 29]. In clinical routine, search for genetic mutations is still debating. Some limitations such as the risk of false positive results have been reported [malignancy associated with RAS mutations is only 74%-87% in different series [30]. In addition, the cut-offs for mutation calling are missing for NGS and RNA from FNAC is often of poor quality for gene analysis [30]. Thus, it will be of great utility to find another marker to apply together with molecular studies to increase molecular analysis performance or, even better, to be used as unique marker able to discriminate benign from malignant cytology with a simple test such as a real-time PCR. In this paper, we showed that Bethesda V–VI nodules (suspicious/malignant) displayed a statistically significant higher expression of PAPPA compared to categories II (benign) and III–IV (indeterminate) with a statistically significant increasing trend from low risk of malignancy categories to high risk. In addition, whatever the cytological category, FNAC malignant at histology had a higher PAPPA expression compared to FNAC benign at histology. Interestingly, a correlation between PAPPA expression and histological results was also observed in the subgroup of III–IV Bethesda nodules. Specifically, PAPPA expression nodules with benign histology were similar to Bethesda II while in those with malignant histology, it was similar to that observed in Bethesda V–VI.

**Fig. 5:**
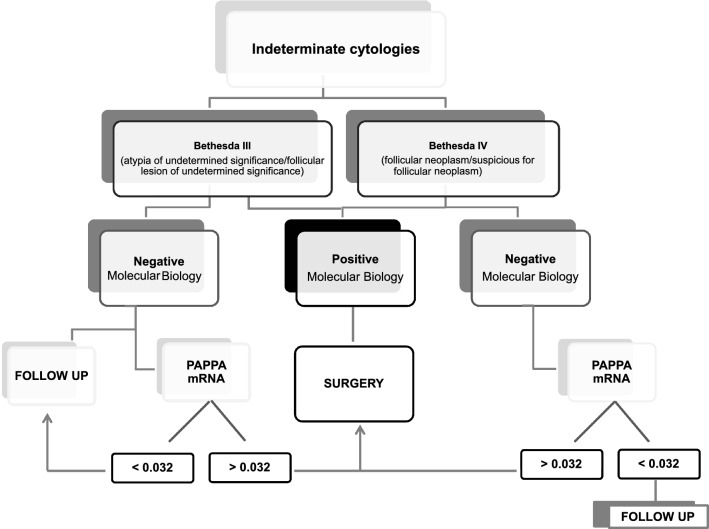
Flow chart of clinical route for indeterminate cytologies combining molecular diagnosis and PAPPA mRNA

We calculated and applied a cut-off able to refine diagnostic accuracy of molecular test alone reaching a specificity and PPV of 100%. These results are encouraging although due to the low number of nodules analyzed (especially in Bethesda V–VI group), it was not possible to drive definitive conclusions and our results need to be replicated in larger series to calculate a robust expression cut-off. However, a hypothetical flow chart to apply in clinical routine for indeterminate cytologies of the combined utility of molecular testing and PAPPA qRT-PCR is depicted in Fig. 5.

The analysis of the IGF1 cascade in the “surgical series” did not showed any difference in IGFBP4, IGF1 and IGF1R mRNA expression in cancer, adenomas and hyperplastic nodules compared to the respective benign counterparts. At difference, a significant reduction of IGFBP4 levels was highlighted in cancers with respect to both adenomas and hyperplastic nodules. These results were similar to those published for ovary cancer compared to normal ovarian tissue in which PAPPA mRNA expression was strongly increased while IGFBP4 was reduced [6].

It is necessary to underline that in our work, we performed only an mRNA expression study and no other functional studies was carried out to analyze the impact of PAPPA on IGF1 cascade. So, further explorations will occur to deep the mechanism(s) by which PAPPA might contribute to the pathogenesis of thyroid cancer.

## Conclusion

In this report, we have shown that PAPPA mRNA expression is increased in thyroid cancer compared to benign lesions in surgical specimen. More importantly, PAPPA mRNA expression is raised in Bethesda V–VI and in indeterminate FNAC malignant at histology, suggesting that PAPPA can be used as potential diagnostic tool to discriminate malignant from benign nodules.
